# Modelling and analysis of penetration resistance of probes in cultivated soils

**DOI:** 10.1371/journal.pone.0280525

**Published:** 2023-01-17

**Authors:** Jing Pang, Xiaojun Lin, Xuwun Zhang, Jiangtao Ji, Lingxin Geng

**Affiliations:** College of Agricultural Equipment Engineering, Henan University of Science and Technology, Luoyang, Henan, China; University of Vigo, SPAIN

## Abstract

At present, the measurement of tillage depth is mainly based on manual measurement, but the manual raking method results in low measurement accuracy and high labor intensity. Due to the complexity of soil, theoretical research on tillage depth is relatively scarce. In order to provide a new research direction and research idea for soil stratification, topsoil was taken as the research object of this paper. The correlation between penetration resistance and penetration depth of a probe in a cultivated soil was studied, and a mathematical model was established. There is a certain similarity between the process of spherical cavity expansion and the process of probe penetration, so we introduced the theory of spherical cavity expansion into the modeling of penetration resistance of the cultivated soil. In this paper, the spherical cavity expansion theory of unsaturated soil was used as the basis for solving the penetration resistance. And the unified strength criterion was employed as a yield condition of the soil to set a stress solution and a displacement solutionin into of the probe penetrating into the elasto-plastic zone of the cultivated soil to determine the model of expansion force. We have carried out indoor tests to revise the expansion force model. Firstly, according to the range of soil density and water content in the field, the soil densities were classified into 1.1×10^3^kg/m^3^, 1.2×10^3^kg/m^3^ and 1.3×10^3^kg/m^3^, and the water contents were divided into 10%, 15% and 20%. In addition, the orthogonal tests were performed at different levels. The soil was put into the barrel, and the probe was inserted into the soil in the barrel at the speed of 8mm/s to determine the test values of the change of the probe penetration resistance with depth. Finally, the expansion force model was fitted with the results of the indoor test, and coefficient B was introduced to express the influence degree of density and water content on the resistance. Coefficient B was substituted into the expansion force model to obtain the penetration resistance model of the cultivated soil. Through the goodness of fit analysis of the penetration resistance model, the results show that the overall average goodness of fit of the penetration resistance modelat was up to 0.871 at different water contents and densities, which was a good fit and could present novel insights into the study relating to soil stratification theory.

## Introduction

Tillage has been found as a vital part of land resources. In agricultural production, it is capable of breaking up the soil, improving soil permeability, and mixing soil and fertilizer uniformly, and it has been the critical and fundamental aspect that has a certain effect on the growth of crops [1–3]. However, the complexity of soils has led to rare theoretical research on tillage depth, and there has been no complete set of mathematical models to describe the correlation between penetration depth and penetration resistance in tilled soils. The theory of circular cavity expansion was initially proposed in 1945, and Vesic [4]. Yu [5, 6] et al, Randolph [7] et al. and Collins [8] et al. have studied cavity expansion theory in depth and divided cavity expansion into column cavity expansion and spherical cavity expansion, which has been extensively applied for static touch tests, pile end resistance studies on engineering static piles and deformation prediction of tunnel envelope. Zhang [9] et al. exploited column bore expansion to analyze the effect of crowding arising from tunnel excavation based on the effect of initial anisotropy of the soil on the mechanical behavior of the soil. These researchers yielded the effective stress solutions for circular tunnel excavation in radial, tangential and vertical directions by Lagrange’s algorithm. Zhou [10] et al. considered the effect arising from the expansion speed on the expansion force and solved the dynamic expansion problem using the modified Cambridge model based on the ODE45 function in Matlab. Liu [11] et al. assumed the soil as a Mohr-Coulomb material and modelled the pile sinking process as a ball-cavity expansion process in semi-infinite soils. To be specific, these researchers adopted Boussinesq solutions corrected for ground stresses to determine pile lateral frictional resistance and pile end resistance. To penetrate hydrostatic piles in saturated clay, Li [12] et al. treated the pile-end action of the penetration process as a spherical cavity expansion process in semi-infinite soils. The yield criterion conformed to the expanded Lade-Duncan, and they derived an elasto-plastic analytical solution. Han [13] et al. considered the sinking pile process column cavity expansion and adopted the Love displacement function and Mohr-Coulomb yield criterion to address the stress and displacement fields in the elastic-plastic zone. Du [14] et al. constructed a logarithmic in-situ soil strength perturbation function to derive the analytical solutions in terms of stress, strain and superporous water pressure for clay column pore expansion under saturated undrained conditions. In general, the study relating to saturated or sandy soils has been simple compared with unsaturated soils, most of which were unsaturated in their natural states. Accordingly, some researchers have conducted studies relating to unsaturated soils. Hu [15] et al. considered the change in soil volume to derive the stress and displacement solutions for ball-cavity expansion in unsaturated soils, while investigating the effect arising from the superconsolidation ratio. Furthermore, Hu [16] et al. considered the effect of the expansion rate of sunken piles in unsaturated soils, while substituting the spherical cavity expansion with a hemispherical cavity to determine the stress displacement of soils after sinking the pile. In accordance with the unified strength theory, Zhao [17] et al. set a unified elastoplastic solution for column cavity expansion for the effect of medium principal stress and matrix suction in unsaturated soils, and they summarized the influence law of medium principal stress, matrix suction and expansion effect. Yang [18] et al. investigated the effect of three different drainage conditions (constant suction, constant moisture content and constant suction on effective stress) on the expansion force for the cavity expansion of unsaturated powder sand. Zhou [19] et al. analyzed the analytical solutions of stresses in unsaturated soils based on different drainage conditions using column-cavity expansion with a uniform strength yield criterion.

The spherical cavity expansion theory adopted in the mentioned papers can be suitable for deep penetration studies, that is, the penetration resistance at the penetration depth of several meters or even tens of meters, whereas it is not suitable for penetration resistance of shallow topsoil (e.g., cultivated soil), and the study focused on the analytical solution of the stress-strain and factors of the ultimate pile end expansion force, without further analyzing the change of resistance during the penetration process. So it is not suitable for direct analysis of penetration resistance of cultivated soil.

At present, there is no unified standard to measure the tillage depth. Generally, manual detection is the main method, or sensors are added to the tillage machines to measure the tillage depth. Manual detection makes the efficiency of the work is low and the intensity is high. When the sensor is affected by the stems and leaves of grass and crops, its stability will also be affected. Therefore, the purpose of this paper is to provide a new research direction and research idea for the discrimination method of tillage depth. The main innovation of this paper is to transform the deep penetration of the spherical cavity expansion theory into the shallow penetration suitable for the cultivated layer, and to establish the stress, strain and displacement solutions for the elastic and plastic zones of unsaturated soils. At the same time, the coefficient B of the influence degree on the penetration resistance was fitted to modify the model, as well as to build a model of the mechanical relationship between the penetration resistance of the probe and the penetration depth. On that basis, this paper attempted to lay a foundation for future theoretical research on the depth of the tillage layer.

## Mechanical model and basic assumptions

**Fig 1:**
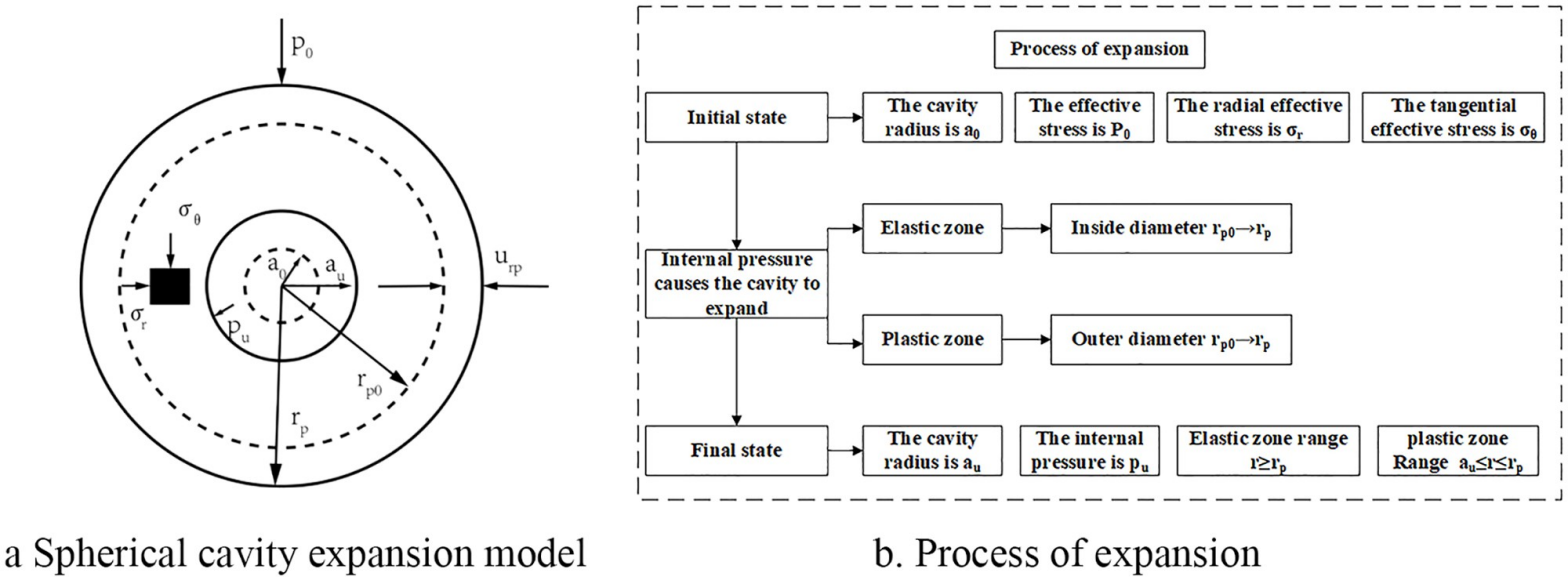
Diagram of spherical cavity expansion theory.

The cavity expansion theory is divided into two basic analysis methods: cylindrical cavity expansion and spherical cavity expansion. There is no strict boundary between different analysis methods. Vesic [4] believes that the prediction of penetration resistance with spherical cavity expansion theory has been accurate enough, so this paper used the spherical cavity expansion theory as the basis for derivation of the penetration resistance model. The spherical cavity expansion model and process of expansion for unsaturated soil were shown in Fig 1. The initial radius of the cavity is expressed as *a*_0_, and the cavity is continuously subjected to internal pressure, so the cavity begins to expand to a radius of *a*_*u*_, corresponding to an internal pressure of *p*_*u*_. The radius of the plastic zone is expanded from *r*_*p*0_ to *r*_*p*_, the displacement is expressed as *u*_*rp*_, the range of the plastic zone is *a*_*u*_ ≤ *r* ≤ *r*_*p*_, and the range of the elastic zone is denoted as *r* ≥ *r*_*p*_. The initial effective stress in the cavity is represented as *p*_0_, and the radial and tangential effective stresses are *σ*_*r*_ and *σ*_*θ*_, respectively.

Since the research object is the cultivated soil in the field, the soil is generally unsaturated. The following assumptions were made according to the expansion state of unsaturated soil [14, 19–22]:

1. Unsaturated soils were assumed as homogeneous and isotropic elastic-plastic bodies.
2. During expanding, the soil was assumed to conform to small strains in the elastic zone, Hooke’s law, and to large strains and a uniform strength criterion in the plastic zone.
3. The magnitude of the initial radial stress was correlated with the depth of the soil. Horizontal initial stresses were defined as [23]: *σ*_*r*0_ = *σ*_θ*0*_ = *K*_0_*σ*_*v*0_ = *K*_0_*gγh*.

Where

*σ*_*r*0_, *σ*_*θ*0_——the horizontal initial radial stress and horizontal initial tangential stress, pa

*σ*_*ν*0_——Vertical stress, pa

*K*_0_——Soil lateral pressure coefficient

*g*——Acceleration of gravity, taken as 9.8N/kg

*γ*——Soil density, kg/m^3^

*h*——Penetration depth, m

## Derivation of cavity expansion theory

### Fundamental equation

Generally, the theory of spherical cavity expansion is solved by combining three sets of basic equations (equilibrium equation, geometric equation and constitutive relation) with failure criterion and boundary conditions.

#### Equilibrium equation

For the expansion of spherical cavity, the soil stress balance equation at any point in the elastic zone is written as:

$$\frac{\partial \sigma_{r}}{\partial_{r}}+2\frac{\sigma_{r}-\sigma_{\theta }}{r}=0$$ (1)

#### Geometric equation

The geometric equation of the small strain theory in the elastic region is presented as:

$$\left\{\begin{array}{l} \varepsilon_{r}=\frac{du}{dr}\\ \varepsilon_{\theta }=\frac{u}{r} \end{array}\right.$$ (2)

Where *ε*_*r*_ and *ε*_*θ*_ denote the radial strain and the tangential strain, respectively; *u* represents the displacement of the soil, and *r* expresses the radius of the soil.

The geometric equation of the theory of large strain in the plastic zone is expressed as:

$$\left\{\begin{array}{l} \varepsilon_{r}=-\text{ln}\frac{dr}{dr_{0}}\\ \varepsilon_{\theta }=-\text{ln}\frac{r}{r_{0}} \end{array}\right.$$ (3)

Where *r*_0_ denotes the initial radius distance of any soil unit within the soil from the center of the cavity; *r* represents the radius of this unit after reaming.

#### Constitutive relation

Under the condition of spherically symmetric, the strain relation in the elastic region, namely the constitutive relation, is denoted as:

$$\left\{\begin{array}{l} \varepsilon_{r}=\frac{1}{E}(\sigma_{r}-2\mu \sigma_{\theta })\\ \varepsilon_{\theta }=\frac{1}{E}((1-\mu )\sigma_{\theta }-\mu \sigma_{r}) \end{array}\right.$$ (4)

Where *E* represents the modulus of elasticity; *μ* denotes the Poisson’s ratio.

#### Stress boundary condition

At *r* ≥ *r*_*p*_, the stress boundary condition of the elastic zone is written as:

$$\left\{\begin{array}{l} \sigma_{r}{|}_{r=rp}=K_{0}\sigma_{vp}\\ \sigma_{r}{|}_{r\to \infty }=K_{0}\sigma_{v0} \end{array}\right.$$ (5)

At *a*_*u*_ ≤ *r* ≤ *r*_*p*_, the stress boundary condition of the plastic zone is expressed as:

$$\left\{\begin{array}{l} \sigma_{r}{|}_{r=ru}=K_{0}\sigma_{vu}\\ \sigma_{r}{|}_{r=rp}=K_{0}\sigma_{vp} \end{array}\right.$$ (6)

#### Yield criterion

Yu’s unified strength theory [24] has a unified mechanical model, unified mathematical modeling equations and unified mathematical expressions that can be applied to different materials and is highly adaptable to unsaturated soils. Thus, the unified strength theory was used as the yield criterion, which is expressed as:

$$\sigma_{r}=M\sigma_{\theta }+\sigma_{0}\;$$ (7)

Among them

$$M=\frac{2(1+b)(1+\text{sin}\;\varphi )+mb(\text{sin}\;\varphi -1)}{\left[2(1+b)-mb\right](1-\text{sin}\;\varphi )}$$

$$\sigma_{0}=\frac{4(1+b)c\;\text{cos}\;\varphi }{\left[2(1+b)-mb\right](1-\text{sin}\;\varphi )}$$

Where *c* denotes the cohesive force of the soil; *φ* represents the angle of friction within the soil; *b* expresses the parameter characterizing the effect of the intermediate principal stress, usually taking values in the range 0 ∼ 1; *m* denotes the intermediate principal stress parameter, under plane strain conditions *m* ≤ 1, when the soil enters the plastic state *m* → 1.

### Theoretical derivation of stress and strain in elastic-plastic zone

#### Elastic zone

According to formula (1) (2) (4) (5), the stress field, strain field and displacement field of the elastic zone are defined as:

$$\left\{\begin{array}{l} \sigma_{r}=K_{0}\sigma_{v0}+(K_{0}\sigma_{vp}-K_{0}\sigma_{v0})\frac{r_{p}^{3}}{r^{3}}\\ \sigma_{\theta }=K_{0}\sigma_{v0}-\frac{1}{2}(K_{0}\sigma_{vp}-K_{0}\sigma_{v0})\frac{r_{p}^{3}}{r^{3}} \end{array}\right.$$ (8)

$$\left\{\begin{array}{l} \varepsilon_{r}=\frac{\left(K_{0}\sigma_{vp}-K_{0}\sigma_{v0}\right)}{4G}\frac{r_{p}^{3}}{r^{3}}\\ \varepsilon_{\theta }=-\frac{\left(K_{0}\sigma_{vp}-K_{0}\sigma_{v0}\right)}{4G}\frac{r_{p}^{3}}{r^{3}} \end{array}\right.$$ (9)

$${\text{u}}_{r}=\frac{\left(K_{0}\sigma_{vp}-K_{0}\sigma_{v0}\right)}{4G}\frac{r_{p}^{3}}{r^{2}}$$ (10)

#### Plastic zone

By substituting (7) into (1), the stress in the plastic zone is yielded as:

$$\sigma_{r}=\;-\frac{\sigma_{0}}{M-1}{\text{+Cr}}^{\frac{2-2M}{M}}\;\;$$ (11)

Where C is the integral constant

Substitute (6) into (11) to get

$$\text{C=(}K_{0}\sigma_{vp}+\frac{\sigma_{0}}{M-1})r_{p}^{\frac{2M-2}{M}}$$ (12)

Combining (7) (11) (12) to get the stress field in the plastic zone

$$\left\{\begin{array}{l} \sigma_{r}=-\frac{\sigma_{0}}{M-1}+\left(K_{0}\sigma_{vp}+\frac{\sigma_{0}}{M-1}\right){\left(\frac{r}{r_{p}}\right)}^{\frac{2-2M}{M}}\\ \sigma_{\theta }=-\frac{\sigma_{0}}{M-1}+\frac{1}{M}\left(K_{0}\sigma_{vp}+\frac{\sigma_{0}}{M-1}\right){\left(\frac{r}{r_{p}}\right)}^{\frac{2-2M}{M}} \end{array}\right.$$ (13)

Plastic strain includes elastic strain and plastic strain

$$\left\{\begin{array}{l} \varepsilon_{r}=\varepsilon_{r}^{e}+\varepsilon_{r}^{p}\\ \varepsilon_{\theta }=\varepsilon_{\theta }^{e}+\varepsilon_{\theta }^{p} \end{array}\right.$$ (14)

Where $\varepsilon_{r}^{e}$ and $\varepsilon_{\theta }^{e}$ represent elastic radial strain and elastic tangential strain, $\varepsilon_{r}^{p}$ and $\varepsilon_{\theta }^{p}$ represent plastic radial strain and plastic tangential strain

Use the associated flow law for the yield process of the soil in the plastic zone

$$\text{d}\varepsilon_{ij}^{p}=d\lambda \frac{\partial g}{\partial \sigma_{ij}}$$ (15)

Where $\varepsilon_{ij}^{p}$ is the plastic strain, *g* is the isoplastic potential surface, and *σ*_*ij*_ is the plastic stress. Substitute (7) into (15) to get

$$\left\{\begin{array}{l} \text{d}\varepsilon_{r}^{p}=d\lambda \\ \text{d}\varepsilon_{\theta }^{p}=-Md\lambda \end{array}\right.$$ (16)

That is

$$\frac{\text{d}\varepsilon_{r}^{p}}{\text{d}\varepsilon_{\theta }^{p}}=-\frac{1}{M}$$ (17)

Integrate both ends of Eq (17) to get

$$\varepsilon_{r}^{p}=-\frac{1}{M}\varepsilon_{\theta }^{p}$$ (18)

#### Elastic—Plastic zone interface

At the elastoplastic interface, substituting Eq (8) into Eq (7), the stress field at the interface is

$$\left\{\begin{array}{l} \sigma_{r}{|}_{r=rp}=\frac{3MK_{0}\sigma_{v0}+2\sigma_{0}}{2+M}\\ \sigma_{\theta }{|}_{r=rp}=\frac{3K_{0}\sigma_{v0}-\sigma_{0}}{2+M} \end{array}\right.$$ (19)

Substituting (19) into (10), the displacement field at the interface is

$${\text{u}}_{r}{|}_{r=rp}=\frac{1}{4G}(\frac{3MK_{0}\sigma_{v0}+2\sigma_{0}}{2+M}-K_{0}\sigma_{v0})r_{p}$$ (20)

From Eqs (2) and (20), the radial and tangential ultimate elastic strains on the interface are

$$\varepsilon_{rp}^{e}=-\varepsilon_{\theta p}^{e}=\frac{1}{4G}(\frac{3MK_{0}\sigma_{v0}+2\sigma_{0}}{2+M}-K_{0}\sigma_{v0})$$ (21)

#### Expansion force model

From the formula (3) (14) (18) (21), the displacement coordination equation of the plastic zone is

$$\text{ln}\frac{d_{r}}{d_{r0}}+\frac{1}{M}\text{ln}\frac{r}{r_{0}}=A(\frac{1}{M}-1)$$ (22)

In

$$\text{A=}\frac{1}{4G}(\frac{3MK_{0}\sigma_{v0}+2\sigma_{0}}{2+M}-K_{0}\sigma_{v0})$$

Solution (22)

$${\text{e}}^{\frac{A(M-1)}{M}}(r^{1+\frac{1}{M}}-r_{0}^{1+\frac{1}{M}})=a^{1+\frac{1}{M}}-a_{0}^{1+\frac{1}{M}}$$ (23)

Where *a* is the cavity radius during the reaming process, and *a*_0_ is the initial radius

According to (23), the total radial and tangential strain in the plastic zone is expressed as:

$$\left\{\begin{array}{l} \varepsilon_{r}=A(1-\frac{1}{M})+\frac{1}{1+M}\text{ln}\left[1+\frac{{\text{e}}^{\frac{A(1-M)}{M}}(a^{1+\frac{1}{M}}-a_{0}^{1+\frac{1}{M}})}{{}^{r_{0}^{1+\frac{1}{M}}}}\right]\\ \varepsilon_{\theta }=-\frac{M}{1+M}\text{ln}\left[1+\frac{{\text{e}}^{\frac{A(1-M)}{M}}(a^{1+\frac{1}{M}}-a_{0}^{1+\frac{1}{M}})}{{}^{r_{0}^{1+\frac{1}{M}}}}\right] \end{array}\right.$$ (24)

From (14) (21) (24), the radial and tangential plastic strains in the plastic zone are denoted as:

$$\left\{\begin{array}{l} \varepsilon_{r}^{p}=\frac{1}{1+M}\text{ln}\left[1+\frac{{\text{e}}^{\frac{A(1-M)}{M}}(a^{1+\frac{1}{M}}-a_{0}^{1+\frac{1}{M}})}{{}^{r_{0}^{1+\frac{1}{M}}}}\right]-\frac{A}{M}\\ \varepsilon_{\theta }^{p}=A-\frac{M}{1+M}\text{ln}\left[1+\frac{{\text{e}}^{\frac{A(1-M)}{M}}(a^{1+\frac{1}{M}}-a_{0}^{1+\frac{1}{M}})}{{}^{r_{0}^{1+\frac{1}{M}}}}\right] \end{array}\right.$$ (25)

On the elastoplastic boundary surface there are

$${\text{e}}^{\frac{A(M-1)}{M}}\left[r_{p}^{1+\frac{1}{M}}-{\left(r_{p}-u_{rp}\right)}^{1+\frac{1}{M}}\right]=a^{1+\frac{1}{M}}-a_{0}^{1+\frac{1}{M}}$$ (26)

Use Taylor’s formula to expand, substituting boundary conditions (20), and ignoring higher-order terms of $\frac{u_{rp}}{r_{p}}$to get

$$\frac{(\frac{3MK_{0}\sigma_{v0}+2\sigma_{0}}{2+M}-K_{0}\sigma_{v0})(1+M){\text{e}}^{\frac{A(M-1)}{M}}}{4GM}{\left(\frac{r_{p}}{a}\right)}^{1+\frac{1}{M}}+{\left(\frac{a_{0}}{a}\right)}^{1+\frac{1}{M}}=1$$ (27)

When *a* → *a*_*u*_,$\frac{a_{0}}{a_{u}}\to 0$,$\frac{r_{p}}{a}\to \frac{r_{p}}{a_{u}}$

(27) is simplified to get the final radius of the plastic zone after expansion:

$$r_{p}={\left[\frac{4GM}{(\frac{3MK_{0}\sigma_{v0}+2\sigma_{0}}{2+M}-K_{0}\sigma_{v0})(1+M){\text{e}}^{\frac{A(M-1)}{M}}}\right]}^{\frac{M}{1+M}}a_{u}$$ (28)

From (13) (28), the expansion force is

$$\begin{array}{l} p_{a}=-\frac{\sigma_{0}}{M-1}+(\frac{3MK_{0}\sigma_{v0}+2\sigma_{0}}{2+M}+\frac{\sigma_{0}}{M-1})\times \\ \;\;\;\;\;\;\;{\left[\frac{(\frac{3MK_{0}\sigma_{v0}+2\sigma_{0}}{2+M}-K_{0}\sigma_{v0})(1+M){\text{e}}^{\frac{A(M-1)}{M}}}{4GM}\right]}^{\frac{2-3M^{2}}{M(1+M)}} \end{array}$$ (29)

## Determination of soil parameters

**Fig 2:**
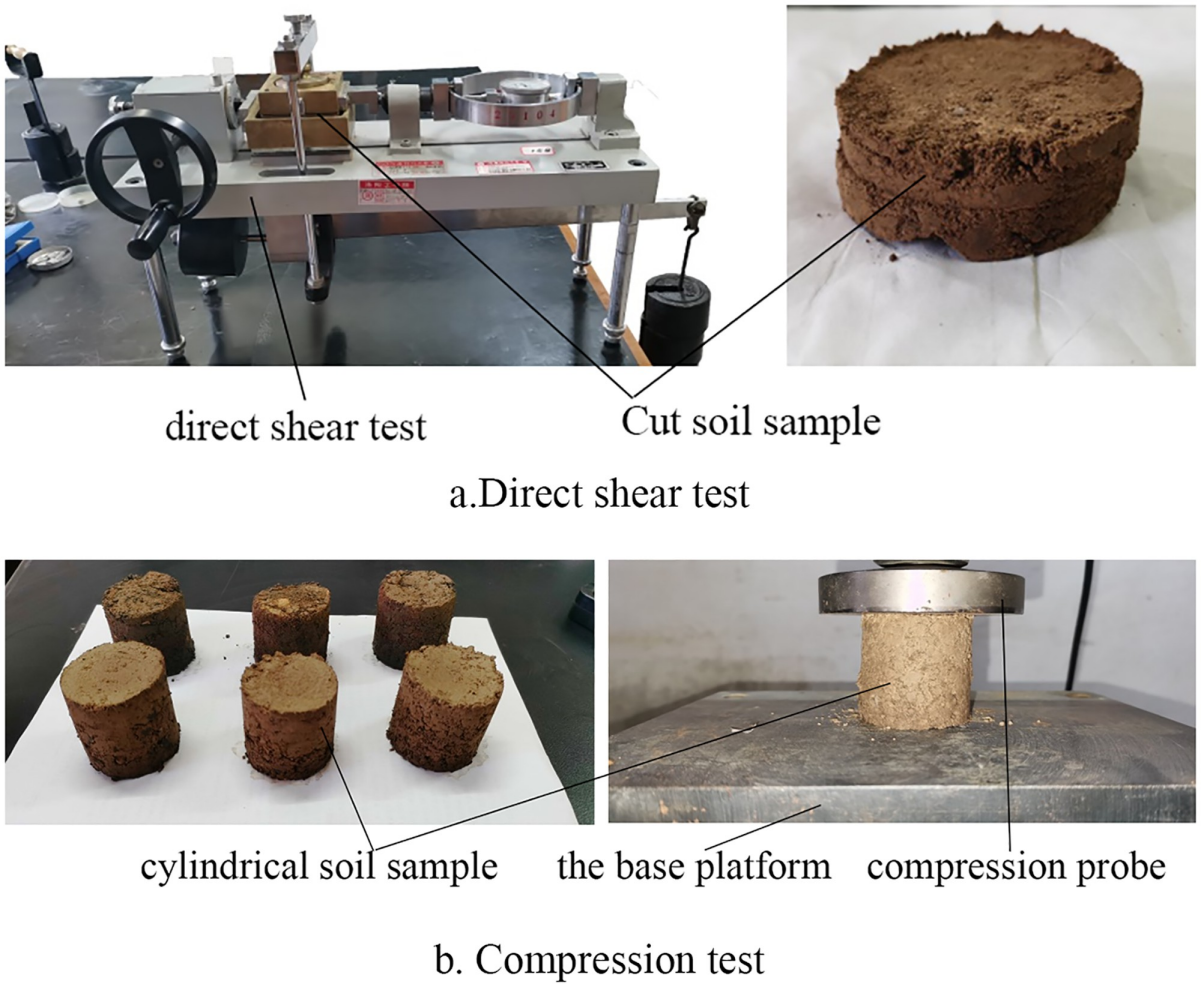
Soil parameter measurement.

In this paper, the soil for the test was selected from a sandy loam from Lingou Village, Mengjin, Henan Province, with 11% of the soil particles have a diameter of less than 0.01mm, 28% have a diameter of 0.01mm-0.05mm, 49% have a diameter of 0.05mm-1mm, and 12% have a diameter of more than 1mm. The soil moisture content was measured by sampling in the field, and the moisture content range was taken as 10%~20%, divided into three levels (10%, 15%, 20%). The parameters (e.g., the angle of internal friction, cohesion and modulus of elasticity of soils) were measured, and the Poisson’s ratio and shear modulus of the soil were calculated by the equations. Before the test, put the soil in the dryer to dry it for more than 48 hours, so that the moisture content of the soil is as close to 0 as possible. Then, according to the moisture content requirements, weigh a certain weight of dry soil and water, spray the water on the dry soil with a watering can, and stir it evenly to make soil samples. The test procedure is illustrated in Fig 2, where (a) represent the direct shear test procedures. The test samples were cut with a ring cutter. The diameter of the samples was 61.8mm and the height was 20mm. The soil was sheared by applying vertical pressures of 100 KPa, 200 KPa, 300 KPa and 400 Kpa. Three tests are conducted under each load condition to calculate the average value of the internal friction angle and cohesion of the soil. (b) represent the procedures to measure the elastic modulus of the soil. Before the test, the dried soil and water were mixed evenly in a certain proportion to make a cylindrical soil sample with a diameter and height of 50mm, which was placed on the platform of DNS02-1KW universal testing machine naturally. The circular indenter with a diameter of 100mm was used to impose load on the cylindrical soil sample at a speed of 0.1mm/s. At the same time, the analysis software reads the data of force (N) -displacement (mm), and stops until the force no longer increases and there is a decreasing trend, at which time the soil sample is crushed. Each test was conducted three times, and the average value of the results was taken. The test results are listed in Table 1.

**Table 1:** Measurement results of soil parameters.

Water content	10%	15%	20%
parameter			
**Internal friction angle /°**	36.19	26	19.51
**Cohesion /pa**	1000	18240	33600
**Elastic Modulus /pa**	2×10^7^	8×10^6^	6.4×10^5^
**Poisson’s ratio**	0.29	0.34	0.4
**Shear modulus /pa**	7.75×10^6^	2.99×10^6^	2.29×10^5^

As indicated by the table above, the angle of internal friction, modulus of elasticity and shear modulus of the soil decreased with the increase in the water content, and the cohesion and Poisson’s ratio of the soil increased with the increase in the water content.

According to the actual influencing factors, the soil structure, density, water content and other factors will affect the experimental results, but for a certain test area, the soil structure will not change greatly, and the influence of water content on soil parameters may be greater than other factors, especially for unsaturated soil. Because unsaturated soil has matric suction, matric suction is related to soil apparent cohesion, which is an important part of the total cohesion. On the other hand, the water film theory of soil suggests that the water in soil contains either free or elastic water films. The elastic water film is surrounded on the surface of soil grain, which has special viscosity, shear resistance and elasticity. In the process of compression and shear of soil samples, only free water can produce lubrication, while bound elastic water has shear resistance, which constitutes the resistance of mutual displacement between soil particles. From this point, the influence of water content on soil parameters is the most important. Therefore, the influence of water content change on soil parameters is mainly measured, and the influence of other factors is ignored.

## Probe penetration test and result analysis

**Fig 3:**
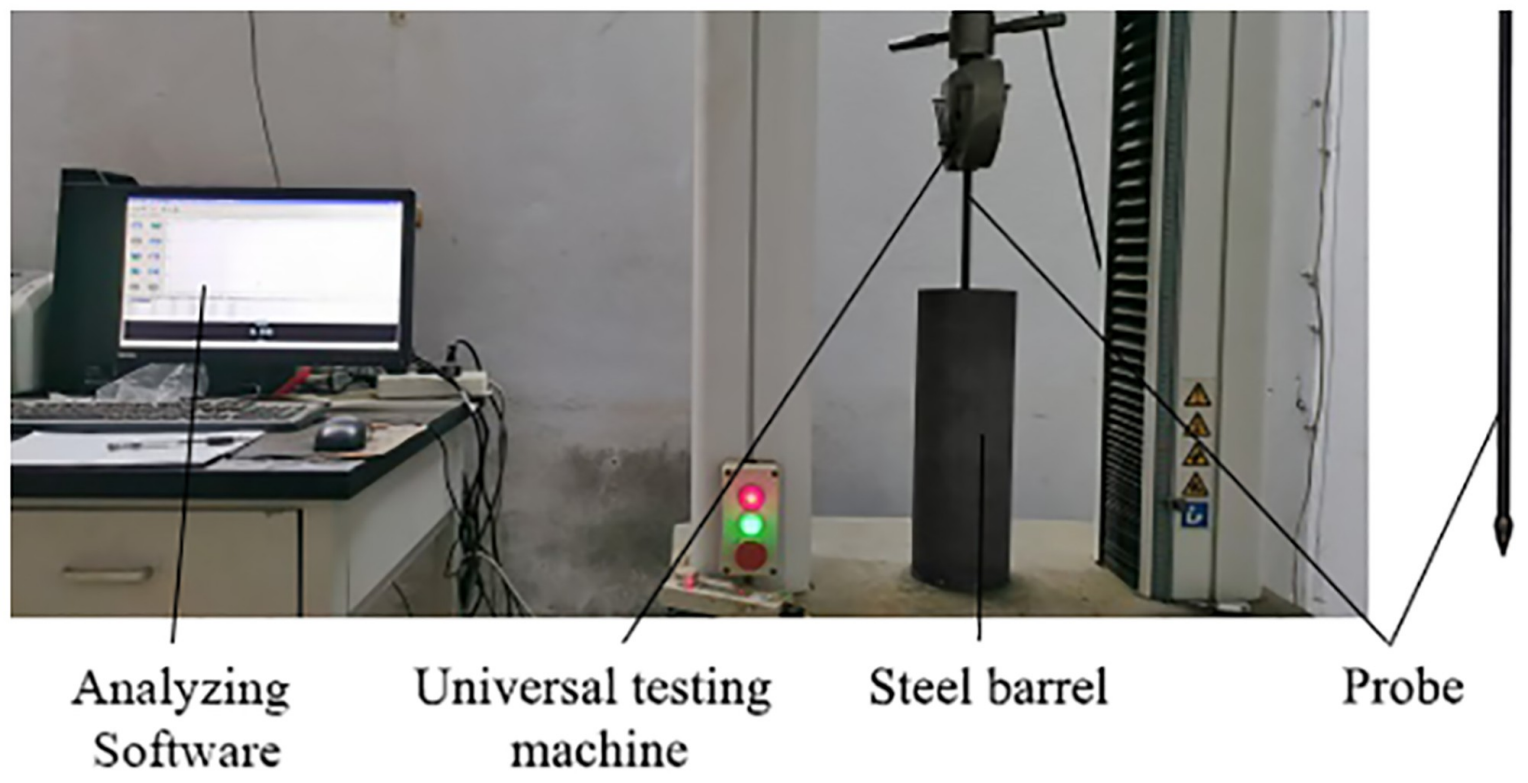
Indoor test of probe penetration into soil.

**Fig 4:**
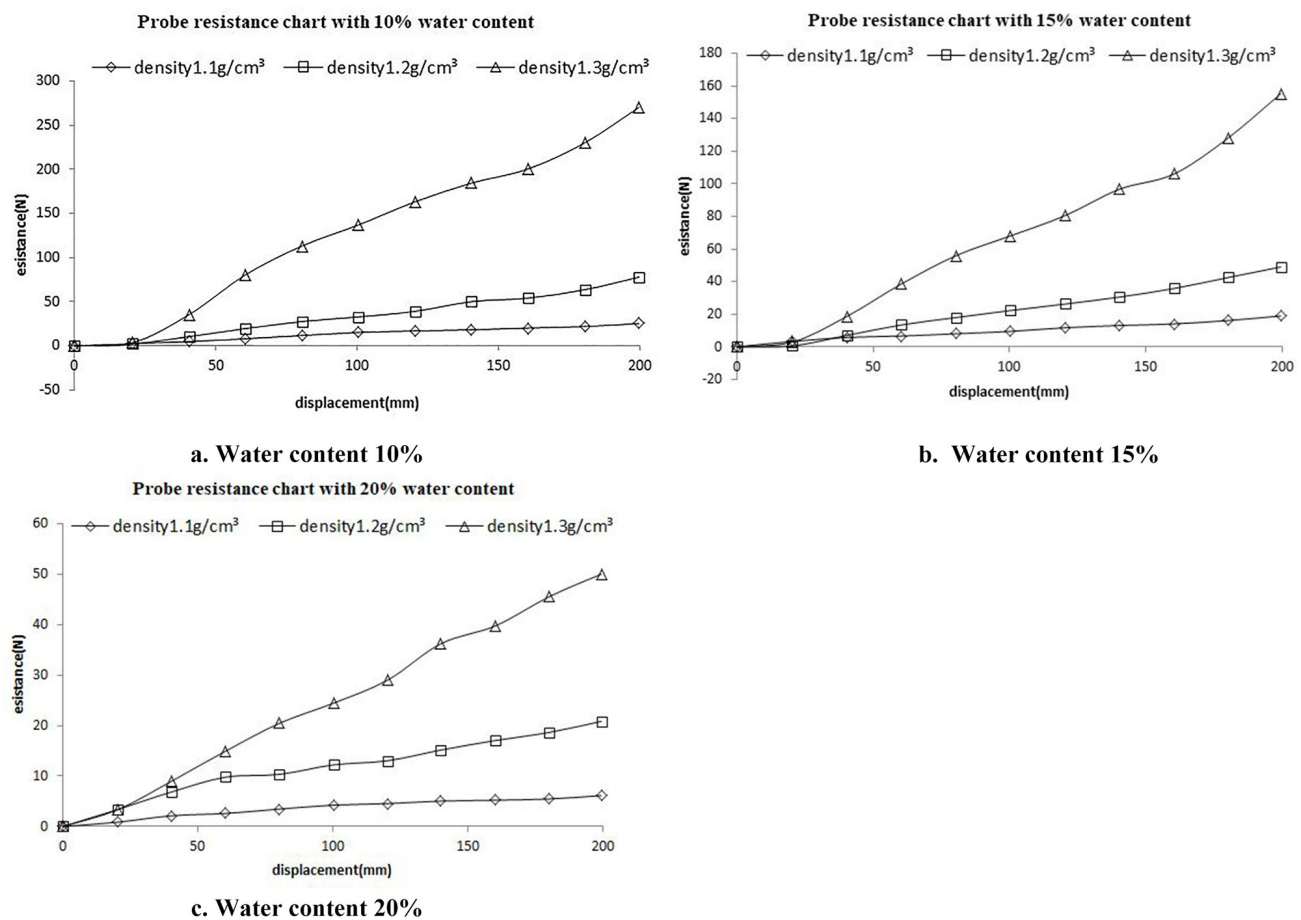
Probe penetration resistance diagram.

To correlate the expansion resistance with the penetration resistance of the probe, a penetration test of the probe was performed indoors. The probe was made of high manganese steel, with a maximum cross-sectional diameter of 14mm as well as an overall probe length of 530mm. The barrel was 124mm in diameter and 400mm in height, and a universal testing machine was used as the test machine. After sampling in the field, it was found that the soil density range was 1.1×10^3^kg/m^3^~1.3×10^3^kg/m^3^, so the soil density was set to 1.1×10^3^kg/m^3^, 1.2×10^3^kg/m^3^ and 1.3×10^3^kg/m^3^ during the test, respectively, and the orthogonal test of probe penetration was performed at 10%, 15% and 20% water content [25–30]. To eliminate errors arising from the barrel wall, the soil depth was set to 300mm, the probe insertion depth was set to 200mm, and the downward movement speed was set to 8mm/s. Calculated the weight of dry soil and water required according to the inner diameter of the barrel, soil height, soil density and water content, mixed the two evenly and put them into the barrel. Shaked the barrel to make the distance between the soil surface and the upper surface of the barrel reach 100mm, so as to ensure that the soil density reached the required value. The barrel filled with soil was placed on the rack of the universal testing machine and installed with the probe. Open the analysis software, adjust the probe height, make sure the bottom of the probe was close to the soil surface, and run the software. The curves of penetration resistance (N) and penetration depth (mm) can be obtained in real time during the test, and the test data can be saved after the test. The indoor test of probe penetration into soil are presented in Fig 3. The test results are presented in Fig 4.

As revealed by the test results, the probe resistance decreased with the increase in the water content and increased with the increase in the density. Moreover, with the increase in the water content, the difference in resistance between different densities was reduced, so a coefficient expressing the degree of influence of density and water content on the resistance B was introduced. The probe resistance in the test was the cone head resistance *q*_*c*_ in *N* and the reaming pressure *p*_*a*_ in *pa*. To correlate the cone head resistance with the reaming pressure and highlight the extent to which density and moisture content have an effect on the resistance, the formula was introduced below:

$${\text{q}}_{\text{c}}={\left(\frac{p_{a}}{10000}\times \frac{\gamma \times 9.8}{1000000}\times \frac{2}{3}\times \pi \times r^{3}\right)}^{\frac{1}{2}}\times h\times 100\times B$$ (30)

Where

*p*_*a*_——Reaming pressure, *pa*

*γ*——Soil density, kg/m3

*r*——The radius of the cone, cm

*h*——Penetration depth, m

Where *B* represents the density coefficient and the moisture content coefficient. To be specific, the density coefficient is expressed as *γ*. The larger the density *γ* is, the greater the penetration resistance will be. It can be seen from Table 1 that the variation trend of soil parameters along with water content. In order to facilitate calculation, parameter *μ*, whose value is relatively easy to calculate, was selected as the water content coefficient. With the increase of water content, the resistance gap between different densities becomes smaller and smaller, so the water content coefficient should also decrease with the increase of water content, which can be expressed by (0.5-*μ*). The soil parameter values under different soil conditions were substituted into Eq (30), and the fitting analysis was carried out with the results of indoor penetration test. It was found that the coefficient B could be expressed by the following formula:

$$B={\left(\frac{\gamma }{1000}\right)}^{12}\times {\left(0.5-\mu \right)}^{\frac{1}{5}}/2.8$$ (31)

Where

*γ*——Soil density, kg/m3

*μ*--Poisson’s ratio

Therefore

$${\text{q}}_{\text{c}}={\left(\frac{p_{a}}{10000}\times \frac{\gamma \times 9.8}{1000000}\times \frac{2}{3}\times \pi \times r^{3}\right)}^{\frac{1}{2}}\times h\times 100\times {\left(\frac{\gamma }{1000}\right)}^{12}\times {\left(0.5-\mu \right)}^{\frac{1}{5}}/2.8$$ (32)

**Fig 5:**
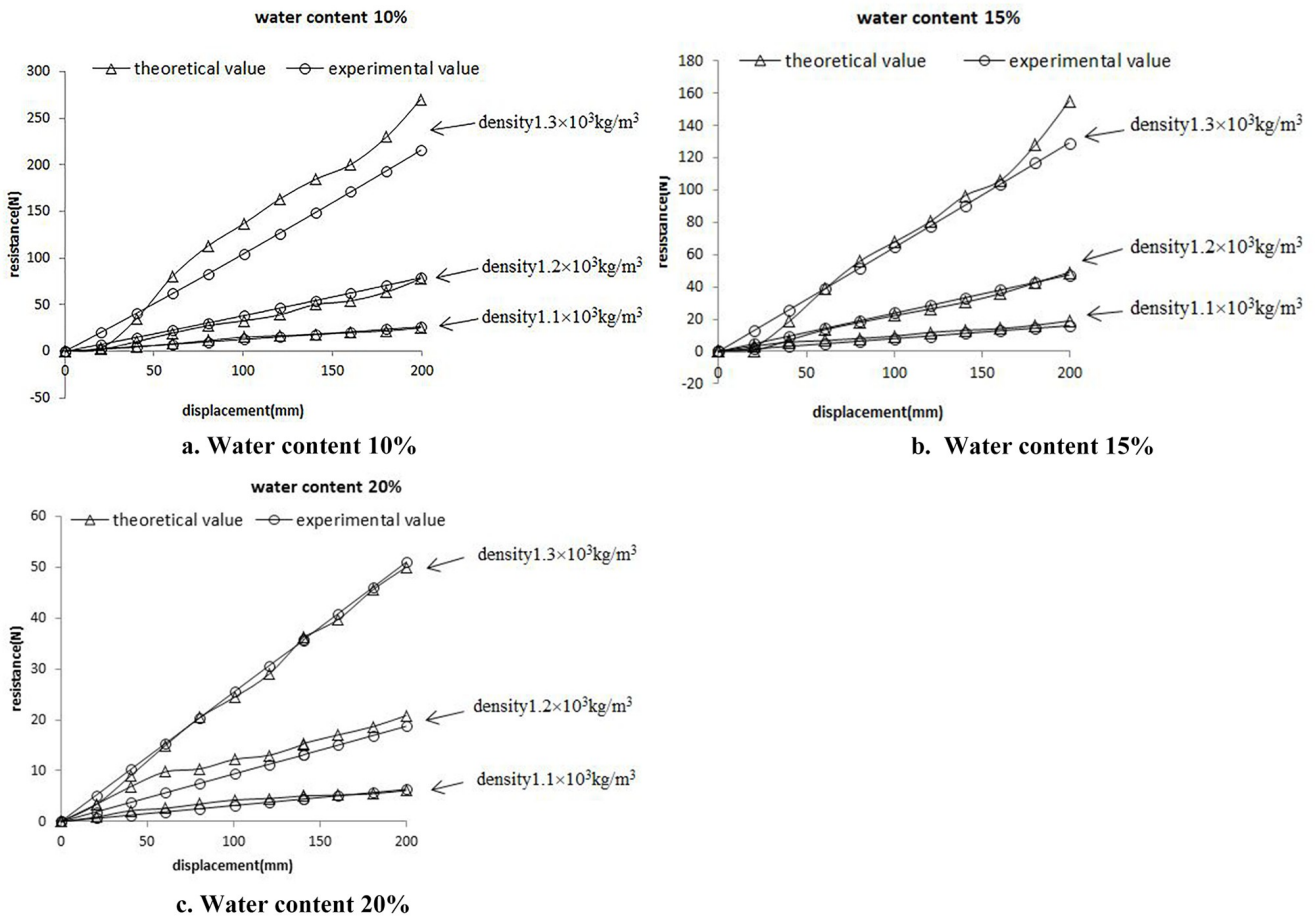
Comparison of theoretical resistance and experimental resistance.

The theoretical cone head resistance values at different water contents and densities were determined by substituting the values into the above equation. The comparison trend between the theoretical and test values is presented in Fig 5, in which the theoretical and test values at different water contents represent the densities 1.3×10^3^kg/m^3^, 1.2×10^3^kg/m^3^ and 1.1×10^3^kg/m^3^, respectively, from top to bottom.

As indicated by Fig 5, the theoretical and experimental values followed roughly the identical path, and R_NL_ [31] was used as a criterion for judging the goodness of fit of the fitted curve of the theoretical values relative to the experimental curve. The closer the fit value ${\overset{⌢}{y}}_{i}$ to *y*_*i*_, the better the fit would be, and the better the estimate of *y*_*i*_ would be by ${\overset{⌢}{y}}_{i}$. R_NL_ combined the residual sum of squares with the relative error; the closer the value to 1, the smaller the difference between the theoretical and experimental values would be. The formula is written as:

$$R_{NL}=1-\sqrt{\frac{\Sigma (y_{i}-{\overset{⌢}{y}}_{i})}{\Sigma y_{i}^{2}}}$$ (33)

Where

*y*_*i*_——Test resistance value

${\overset{⌢}{y}}_{i}$——Theoretical resistance value

Based on the comparison of the goodness of fit of the experimental and theoretical values, the goodness of fit for the nine cases with different moisture contents and densities is listed in Table 2.

**Table 2:** Goodness of fit of theoretical resistance value.

Soil condition	goodness of fit
Water content 10%, density 1.1×10^3^k g/m^3^	0.924
Water content 10%, density 1.2×10^3^k g/m^3^	0.872
Water content 10%, density 1.3×10^3^k g/m^3^	0.800
Water content 15%, density 1.1×10^3^k g/m^3^	0.828
Water content 15%, density 1.2×10^3^k g/m^3^	0.918
Water content 15%, density 1.3×10^3^k g/m^3^	0.885
Water content 20%, density 1.1×10^3^k g/m^3^	0.829
Water content 20%, density 1.2×10^3^k g/m^3^	0.816
Water content 20%, density 1.3×10^3^k g/m^3^	0.963
average value	0.871

As indicated by the table, the goodness of fit for the theoretical values of probe resistance is higher than 0.8 under different water contents and densities, which could be considered a good fit, and the average goodness of fit reaches 0.871. This proves that under the same soil conditions, the penetration resistance model can completely replace the probe penetration trough test, reducing the workload of researchers to a certain extent. Most importantly, this study can provide a theoretical basis for the actual tillage depth analysis in the field. If the probe is inserted into the tilled soil in the field, the actual tillage depth can be determined by analyzing the curve of the probe’s penetration resistance and penetration depth, and combining with the curve trend of the penetration resistance model.

## Comparative analysis of models

In order to prove the applicability of the model proposed in this paper to shallow penetration of soil, the model was compared with the existing relevant research models [17, 19]. The basic physical characteristics of soil are as follows: soil moisture content 15%, density 1.2g/cm^3^, internal friction Angle φ = 26°, cohesibility C = 18240Pa, Poisson’s ratio μ = 0.34, shear modulus E = 2.99×10^6^Pa, b = 0.5, m = 0.5, P_0_ = 6.5KPa.

**Fig 6:**
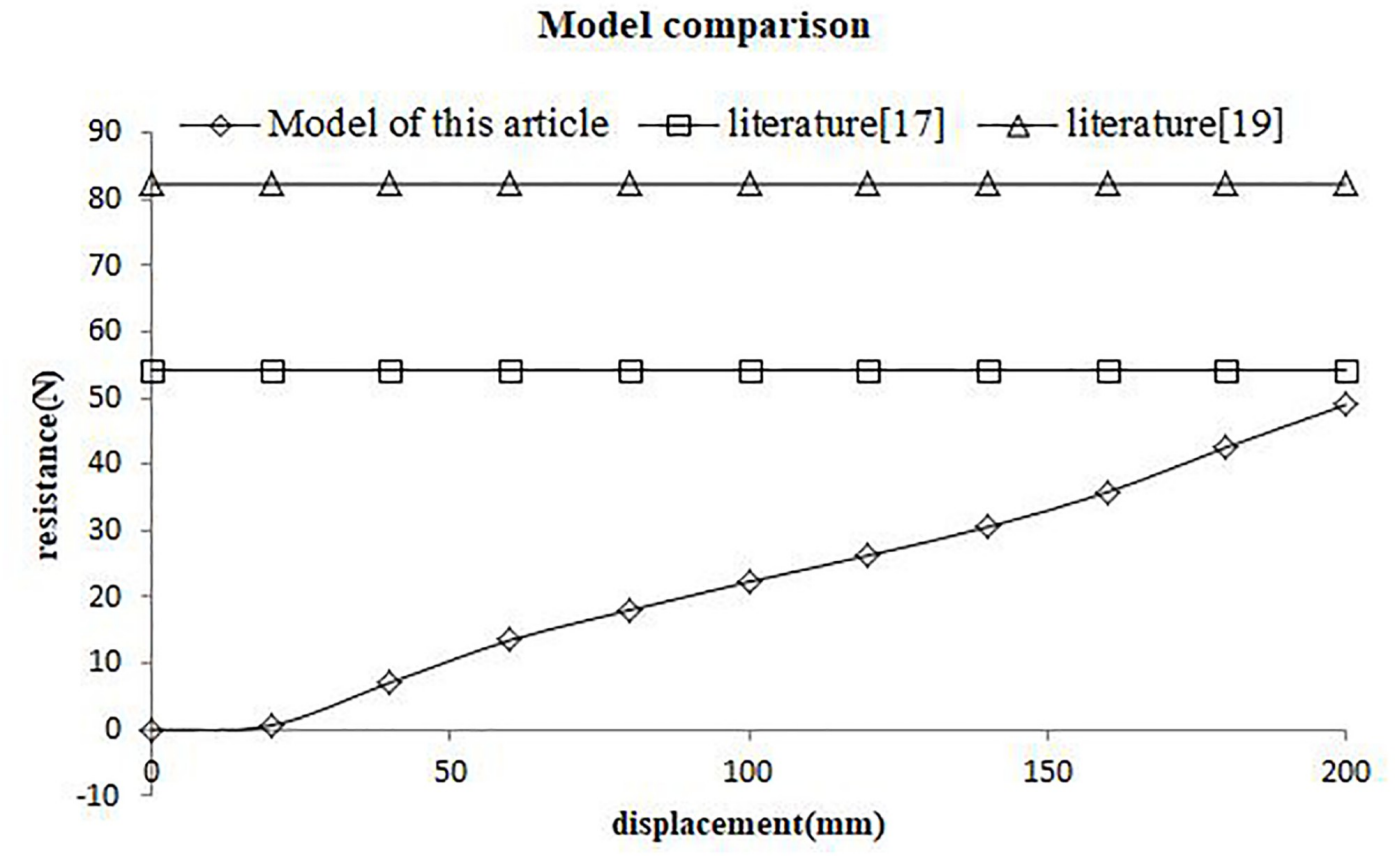
Model comparison results.

It can be seen from Fig 6 that with the increase of depth, the penetration resistance of existing relevant models does not change significantly in the direction of depth, while the penetration resistance of this model presents an obvious upward trend with the increase of depth. This is because the existing relevant research models are all about the deep penetration problem, that is, the penetration at the depth of several meters or even tens of meters. Small changes in the depth direction have little impact on their research, so they locate the horizontal initial stress P_0_ at different depths to a constant value. This model is mainly aimed at the shallow penetration problem, the depth gap is only tens of centimeters. In order to enlarge the resistance gap of tens of centimeters, the horizontal initial stress P_0_ in this paper is related to the depth and will change with the depth. At the same time, the fitting coefficient B further enlarges the gap between penetration resistance values, so as to achieve the research of penetration resistance suitable for shallow soil.

## Conclusion

1. In this paper, a model of spherical pore expansion in cultivated soils was built. Next, the probe penetration process was modelled as a process of small strain in the elastic zone and large strain in the plastic zone of the soil. Subsequently, the unified strength theory was employed as the yield criterion for unsaturated soils, thereby leading to the expansion pressure of the penetration.
2. As revealed by the measurements of soil parameters, the angle of internal friction, modulus of elasticity and shear modulus decreased with the increase in the water content, and the cohesion and Poisson’s ratio of the soil increased with the increase in the water content. According to the probe penetration tests, the resistance of the probe decreased with the increase in the water content and increased with the increase in the density, and the difference in resistance between densities was less significant with the increase in the water content.
3. By linking the reaming pressure with the penetration resistance and introducing the density coefficient and water content coefficient based on the analysis of the test results, a theoretical model was built for the change of the resistance of the probe with depth under different soil conditions of the tillage layer, and the goodness of fit of the model was verified through comparative analysis, which had certain reasonable feasibility.

## Supporting information

S1 File (XLSX)
